# A Single LC-MS/MS Analysis to Quantify CoA Biosynthetic Intermediates and Short-Chain Acyl CoAs

**DOI:** 10.3390/metabo11080468

**Published:** 2021-07-21

**Authors:** Anthony E. Jones, Nataly J. Arias, Aracely Acevedo, Srinivasa T. Reddy, Ajit S. Divakaruni, David Meriwether

**Affiliations:** 1 Department of Molecular and Medical Pharmacology, David Geffen School of Medicine, University of California, Los Angeles, 650 Charles E Young Dr. South, Los Angeles, CA 90095, USA; aejones@mednet.ucla.edu (A.E.J.); natalyarias@mednet.ucla.edu (N.J.A.); aacevedo@mednet.ucla.edu (A.A.); sreddy@mednet.ucla.edu (S.T.R.); 2 Department of Medicine, Division of Cardiology, David Geffen School of Medicine, University of California, Los Angeles, 10833 Le Conte Avenue, Los Angeles, CA 90095, USA; 3 Department of Medicine, Division of Digestive Diseases, David Geffen School of Medicine, University of California, Los Angeles, 10833 Le Conte Avenue, Los Angeles, CA 90095, USA

**Keywords:** LC-MS/MS, CoA biosynthesis, short-chain acyl CoAs, mitochondria, etomoxir

## Abstract

Coenzyme A (CoA) is an essential cofactor for dozens of reactions in intermediary metabolism. Dysregulation of CoA synthesis or acyl CoA metabolism can result in metabolic or neurodegenerative disease. Although several methods use liquid chromatography coupled with mass spectrometry/mass spectrometry (LC-MS/MS) to quantify acyl CoA levels in biological samples, few allow for simultaneous measurement of intermediates in the CoA biosynthetic pathway. Here we describe a simple sample preparation and LC-MS/MS method that can measure both short-chain acyl CoAs and biosynthetic precursors of CoA. The method does not require use of a solid phase extraction column during sample preparation and exhibits high sensitivity, precision, and accuracy. It reproduces expected changes from known effectors of cellular CoA homeostasis and helps clarify the mechanism by which excess concentrations of etomoxir reduce intracellular CoA levels.

## 1. Introduction

Coenzyme A (CoA) is an obligatory co-factor in all organisms [1]. It is involved in several aspects of mammalian cellular metabolism including the Krebs cycle, oxidation of fatty acids and branched chain amino acids, as well as synthesis of fatty acids and sterols. CoA acts as an acyl group carrier forming thioester linkages with organic acids to yield acyl CoAs (e.g., acetyl CoA or palmitoyl CoA) [1]. The formation of a CoA thioester serves multiple functions. The large free energy of hydrolysis of the thioester bond serves as a means to ‘charge’ or ‘activate’ the adjoining acyl group for further metabolism. Additionally, formation of CoA esters can aid in subcellular metabolite compartmentation. These acyl CoA esters are used as building blocks for biosynthetic reactions, substrates for post-translational modifications, or energy substrates oxidized to ultimately produce ATP [2].

In mammals, *de novo* synthesis of CoA begins with the cellular uptake of pantothenate (vitamin B_5_) via the sodium-dependent multivitamin transporter (SMVT) [3,4]. Five enzymatic reactions convert pantothenate into CoA. Pantothenate is first phosphorylated by pantothenate kinase (PANK), the primary rate-controlling step in CoA synthesis [5]. The subsequent conjugation with cysteine followed by decarboxylation results in 4′-phosphopantetheine. Lastly, 4′-phosphopantetheine is converted to CoA by the bifunctional enzyme Coenzyme A synthase (COASY). An adenylyl group from ATP is first transferred to 4′-phosphopantetheine to form dephospho-CoA, and followed by phosphorylation of dephospho-CoA to form unesterified (“free”) CoA [1].

Free CoA can be used to activate carboxylic acids of differing chain lengths through the action of various CoA ligases [6,7]. Disruption of CoA biosynthesis or short-chain acyl CoA metabolism can result in pathological defects. For example, altering levels of short-chain acyl CoAs disrupts hepatic metabolic homeostasis [8], and certain forms of neurodegeneration are caused by mutations in enzymes responsible for CoA synthesis [9,10]. Short-chain acyl CoAs also have additional roles in cell biology beyond energy metabolism, such as in post-translational modifications. In macrophages, for example, increased acetyl CoA production via ATP citrate lyase (ACLY) is associated with histone acetylation and the epigenetic changes observed during the anti-inflammatory activation with interleukin-4 (IL-4) [11]. Further work has demonstrated that intracellular CoA levels are associated with IL-4-driven macrophage activation [12]. However, the precise mechanism by which this occurs, as well as which short-chain CoA esters are similarly associated with the IL-4 response, remains unclear. A single assay for measuring CoA biosynthetic intermediates as well as short-chain acyl CoA species is therefore important to better understand CoA homeostasis in response to physiologically relevant stimuli such as macrophage activation.

Several techniques are available to measure short-chain acyl CoAs. These include fluorescence-based enzymatic assays, high performance liquid chromatography (HPLC), gas chromatography-mass spectrometry (GC-MS), and HPLC coupled with tandem mass spectrometry-based (LC-MS/MS) assays. Of these, LC-MS/MS-based methods offer the highest selectivity and sensitivity [13,14].

Although several published procedures detail the quantification of acyl CoAs of different chain lengths [15,16,17,18,19,20,21,22], none currently present a single method for the extraction and analysis of both short-chain acyl CoAs and intermediates in the CoA biosynthetic pathway. A primary challenge for this lies in the preparation of the biological sample. Many LC-MS/MS assays use halogenated carboxylic acids or oxo-acids for deproteinizing the sample (e.g., trichloroacetic acid) [23]. Solid phase extraction (SPE) is then used to purify acyl CoAs and remove the deproteinizing agent before LC-MS/MS analysis [18,19,20,22]. Mechanistically, SPE operates by retaining the acyl CoA species on the solid phase sorbent while the aqueous phase containing the deproteinization agent is discarded as waste [24]. While SPE efficiently binds relatively hydrophobic acyl CoAs (e.g., propionyl CoA, isovaleryl CoA, etc.), it does not efficiently retain relatively hydrophilic CoA biosynthetic pathway intermediates such as dephospho-CoA and pantothenate. It is therefore difficult to use SPE when measuring both acyl CoAs and CoA biosynthetic intermediates. Their divergent polarities also create difficulty in developing a single LC-MS/MS method. Relatively hydrophobic short-chain acyl CoAs exhibit the best chromatography when using traditional C18 columns under reverse phase conditions [6]. Under those same conditions, however, relatively hydrophilic species including pantothenate, dephospho-CoA, and free CoA exhibit poor chromatographic peak shape and retention [17,25].

Here we present a single sample preparation and LC-MS/MS method that allows for the extraction and quantification of both short-chain acyl CoAs as well as the CoA biosynthetic intermediates pantothenate and dephospho-CoA. We use 5-sulfosalicylic acid (SSA) for sample deproteinization, as this compound obviates the need for removal by SPE prior to LC-MS/MS analysis. This therefore retains a significant amount of pantothenate and dephospho-CoA from biological samples that would otherwise be lost following SPE-based purification. We overcome the challenge of poor chromatographic separation between the various species by carefully controlling the pH, thereby minimizing the charge on the competing positively and negatively polarizable moieties of the CoA backbone. We then further suppress and mask remaining polarization and charge with ion-pairing chromatography. When combined with an ultra-high performance liquid chromatography (UHPLC) C18 column with high theoretical plates, we observe stable and symmetric peak shape with good resolution across a range of analytes. We demonstrate that this method is able to reproduce findings generated using other LC-MS/MS methods and also helps to reveal the mechanism by which excess concentrations of the carnitine palmityoltransferase-1 (CPT-1) inhibitor etomoxir decreases intracellular CoA levels in bone marrow-derived macrophages (BMDMs).

## 2. Results

### 2.1. The MRM MS/MS Method Can Detect Both CoA Biosynthetic Intermediates and Short-Chain Acyl-CoAs

**Figure 1:**
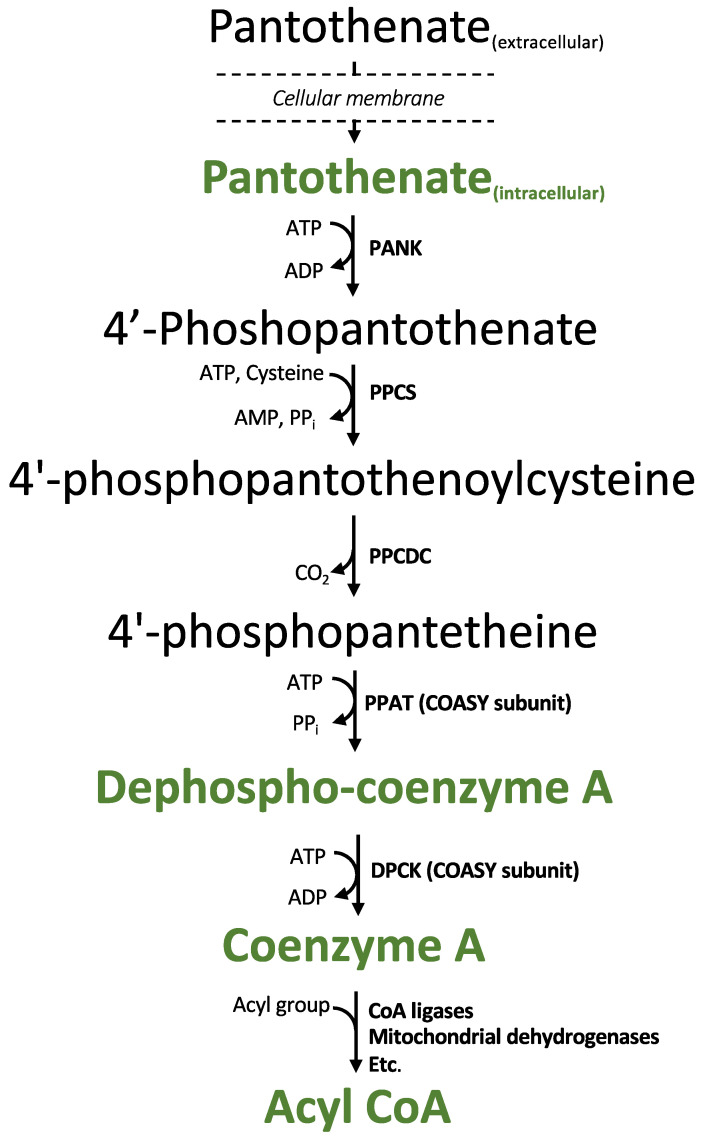
The CoA biosynthetic pathway: the metabolites detected by the method detailed in this manuscript are highlighted in green. Abbreviations: PANK = pantothenate kinase; PPCS = phosphopantothenoylcysteine synthase; PPCDC = phosphopantothenoylcysteine decarboxylase; PPAT = phosphopantetheine adenylyl transferase; and DPCK = dephosphocoenzyme A kinase. The bifunctional enzyme Coenzyme A synthase (COASY) is comprised of PPAT and DPCK.

We initially optimized the mass spectrometry to ensure robust detection of both short-chain acyl CoAs as well as pantothenate and dephospho-CoA (Figure 1, Figure S1).

**Figure 2:**
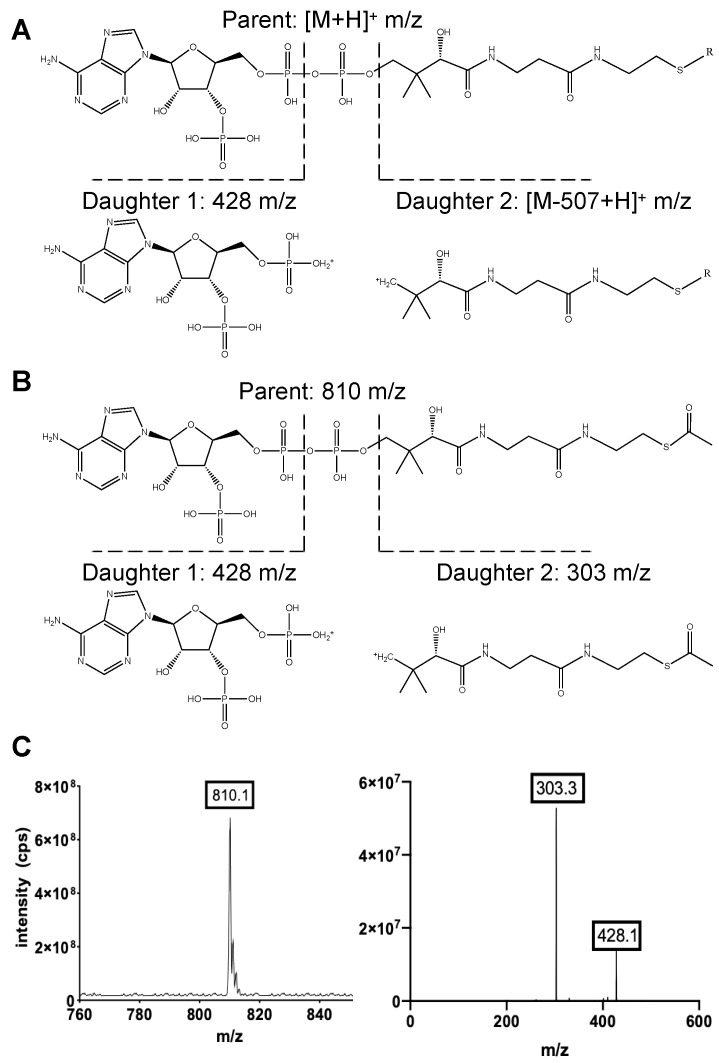
The common MS/MS fragmentation pattern for all CoA species: (**A**) The CoA portion of all CoA esters fragments during MS/MS at the 3′-phosphate-adenosine-5′-diphosphate. This cleavage gave rise to a daughter ion equal to [M − 507 + H]^+^ m/z (right). “M” is the molecular mass of the starting compound. The phosphate-adenosine portion also fragments between the 5′-diphosphates and gave rise to a daughter equal to 428 m/z (left). (**B**) Acetyl CoA has a parent m/z of 810 with a predicted fragmentation pattern for daughter ions of 428 m/z and 303 m/z. (**C**) The MS1 parent and predicted MS2 daughter ions for acetyl CoA were detected following direct infusion of 1 μg/mL acetyl CoA into the mass spectrometer.

To optimize the MS detection of short-chain acyl CoAs, we prepared 1 μg/mL solutions of each acyl CoA standard in the SSA extraction solution (see Methods) and these standards were directly infused into the mass spectrometer for MS/MS analysis. We focused on optimizing the detection of short-chain acyl CoAs with the MS operated in positive mode, as previous studies demonstrated that they are more efficiently ionized under these conditions [15]. We observed the characteristic fragmentation pattern for all acyl CoA species. The CoA portion was cleaved at the 3′-phosphate-adenosine-5′-diphosphate during positive mode MS/MS. This fragmentation gave rise to a neutral loss of 507 atomic mass units (amu) together with a daughter ion equal to a mass-to-charge ratio (m/z) of [M − 507 + H]^+^, where “M” is the molecular mass of the initial compound. Additionally, the phosphate-adenosine portion of each acyl CoA species fragmented between the 5′-diphosphates and gave rise to a daughter equal to 428 m/z [26] (Figure 2A). For example, acetyl CoA has a monoisotopic mass of 809.1 and therefore gives rise to an MS1 parent of 810.1 m/z. Following fragmentation, daughter ions of 303 m/z (indicating cleavage at the 3′-phosphate-adenosine-5′-diphosphate) and 428 m/z (indicating fragmentation between the 5′ diphosphates) were both apparent (Figure 2B,C). We used multiple reaction monitoring (MRM) to both identify and quantify each acyl CoA species detected with this method. For each acyl CoA, two separate transitions were monitored: (i) [M + H]^+^ fragmenting to [M − 507 + H]^+^ m/z was used for quantitation, and (ii) [M + H]^+^ fragmenting to 428 m/z was used for qualitative identification (Table 1).

After tuning for efficient detection of acyl CoAs, we optimized the detection of CoA synthetic intermediates. Dephospho-CoA, similar to acyl CoAs, was efficiently detected in positive mode. The quantitative and qualitative MRM transitions of dephospho-CoA were determined empirically during tuning (Table 1). Initial efforts to optimize the detection of pantothenate in the positive mode MRM were unsuccessful, and much greater sensitivity was observed using the negative mode MRM (Figure S2). As a result, the negative mode MRM was used for pantothenate, while the positive mode MRM was used for all other analytes. Additional settings for each transition including declustering potential (DP), entrance potential (EP), collision energy (CE), and cell exit potential (CXP) were optimized during tuning with reference standards (Supplemental Table S1). Source settings of temperature, ion spray voltage, and gas flows were likewise optimized during method development (see Methods).

### 2.2. Ion-Pairing UHPLC Chromatography Produces Well-Separated Peaks for CoA Biosynthetic Intermediates and Short-Chain Acyl CoAs

We next developed the chromatography parameters for CoA biosynthetic intermediates and short-chain acyl CoAs. Like other phosphorylated organic molecules, CoA can be a difficult structure to resolve using common chromatographic techniques [25]. Its three phosphate groups are polar and capable of negatively ionizing at higher pH, resulting in poor affinity for the solid phase of common reverse phase columns like C18. Simultaneously, the adenine moiety can serve as a Bronsted–Lowry base and acquire a proton at a pH below 4.0. Moreover, these polar or charged groups are offset by acyl groups of increasing non-polarity as the length of the acyl chain increases.

We first tested a standard HPLC C18 column using an acetonitrile gradient containing common modifiers such as 0.1% (*v*/*v*) formic acid or 5 mM ammonium acetate. Formic acid produced extremely poor chromatography (not shown), thus ammonium acetate was used as the modifier and proton source. Unmodified ammonium acetate still produced considerable peak tailing, thus the pH was carefully adjusted with acetic acid to pH 5.6. This value was selected in order to be above the pKa of the adenine NH_3_^+^ (4.0) but below the pKa of the secondary phosphate (6.4) [27]. While the chromatography of the CoA species was improved, it still exhibited poor peak shape. The poor results at pH 5.6 are likely explained by the primary phosphate of the CoA backbone retaining a negative charge and pantothenate remaining as an anion.

**Figure 3:**
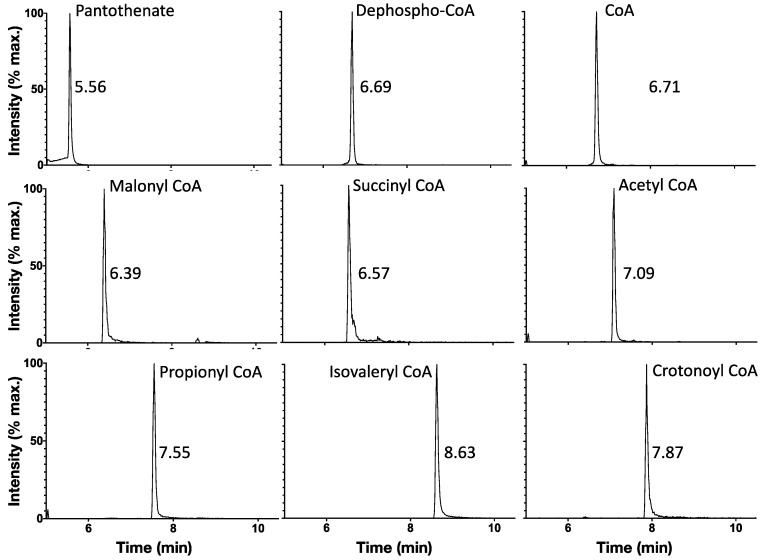
Ion-pairing UHPLC chromatography produces well-separated peaks for CoA biosynthetic intermediates and short-chain acyl CoAs. All eight compounds together with the internal standard (crotonoyl CoA) were injected at 1 μg/mL into the LC-MS/MS. Retention times are listed to the right of each peak. Liquid chromatography consisted of a reverse-phase gradient with solvents A & B (see Table 2) in addition to a Phenomenex Kinetex, 2.6 mm × 150 mm UHPLC C18 column. The quantitative MRM channel of each species was isolated and retention times were determined.

The remaining chromatographic difficulties were resolved by ion pairing chromatography combined with use of a UHPLC C18 column with high theoretical plates. N,N-dimethylbutylamine (DMBA) is a tertiary amine that acquires a proton below pH 10.0 [28] and has been used as an ion pairing agent to improve the chromatography of phosphate-containing compounds such as oligonucleotides [29]. DMBA has also been used to improve the chromatography of short-chain acyl CoA species such as malonyl CoA [17]. It was therefore added to the ammonium acetate solution (solvent A; Table 2) and a Phenomenex Kinetex ultra-high performance liquid chromatography (UHPLC) C18 column was used. The resulting chromatography produced well-separated peaks with minimal tailing for both CoA biosynthetic intermediates and short chain acyl CoAs (Figure 3).

Importantly, dephospho-CoA appears to form in the electrospray ionization (ESI) source from acetyl, propionyl, and isovaleryl CoA (Figure S3). It is therefore crucial to carefully distinguish the peaks and retention times associated with dephospho-CoA itself from those resulting from these other analytes in order to avoid erroneous detection of dephospho-CoA.

### 2.3. The Method Displays a Linear Detection of Analytes across a Wide Concentration Range with Sensitive Lower Limits of Detection and Quantitation

**Figure 4:**
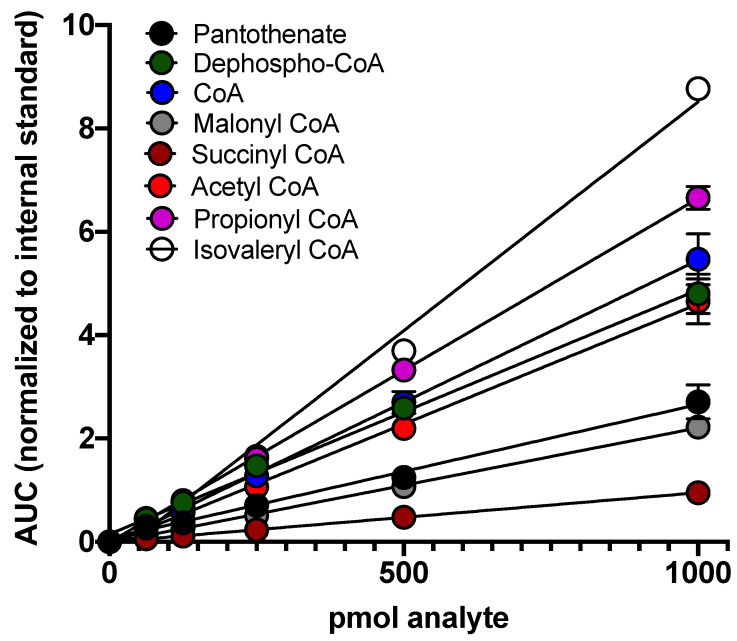
The LC-MS/MS method demonstrates a linear detection of analytes across a wide concentration range: standards were spiked into 200 μL of extraction solution and analyzed via LC-MS/MS. Calibration curves were generated by plotting the area under the curve (AUC) for each analyte (normalized to the internal standard) against the amount of analyte contained in 200 μL of extraction solution. Data are presented as mean ± standard error of the mean (S.E.M.) for *n* ≥ 3 technical replicates.

The sensitivity and linearity of the LC-MS/MS method were determined by spiking a range of reference analytes into the sample extraction solution in addition to the internal standard crotonoyl CoA. Calibration curves were fit by linear regression with 1/x weighting (Figure 4).

The lower limit of detection (LLOD) for each analyte was determined as the lowest concentration that produced a signal at least five times greater than that of the corresponding noise floor. The lower limit of quantitation (LLOQ) was determined as the lowest concentration in the calibration curve for which the curve-calculated concentration was within 20% of its nominal concentration. The linearity of each calibration curve was determined as the coefficient of correlation *r*. For each analyte, the LC-MS/MS yielded sensitive LLOD and LLOQ with *r* ≥ 0.95 (Table 3).

### 2.4. Extraction with 2.5% SSA Is Suitable for Analysis of Acyl CoAs and CoA Biosynthetic Intermediates

Following refinement of the LC-MS/MS settings, we next determined an appropriate method to extract acyl CoAs and CoA biosynthetic intermediates from biological material. We first evaluated common acyl CoA extraction procedures to test their capacity to recover dephospho-CoA and pantothenate. Acyl CoAs are commonly extracted with halogenated forms of acetic acid [e.g., trichloroacetic acid (TCA) and trifluoroacetic acid (TFA)] followed by solid phase extraction (SPE) to remove the deproteinizing agent. After SPE purification, acyl CoAs are resuspended in a solvent favorable for LC-MS/MS analysis such as water or SSA [18,19,22].

To control for variabilities in the sample extraction procedure, we again used crotonoyl CoA as an internal standard. Complete precision under all possible conditions for a given CoA species or biosynthetic intermediate requires a matched, isotopically labeled reference species. This can be accomplished by SILEC labeling [21], which exploits the lack of *de novo* pantothenate synthesis in mammalian and insect cells to generate such standards with the provision of labeled pantothenate in cell culture medium. Although this technique is the gold standard for such assays, it can be time and cost prohibitive for almost all non-specialist labs. We therefore used crotonyl CoA as an inexpensive and amenable standard for this straightforward method. Figure S4 illustrates that endogenous crotonyl CoA levels range from less than 1% (cultured cells) to an upper bound of 3% (5 mg liver tissue) of the internal standard. As such, endogenous changes in crotonyl CoA levels would only introduce a marginal error when quantifying short-chain CoAs and CoA biosynthetic intermediates under most conditions.

**Figure 5:**
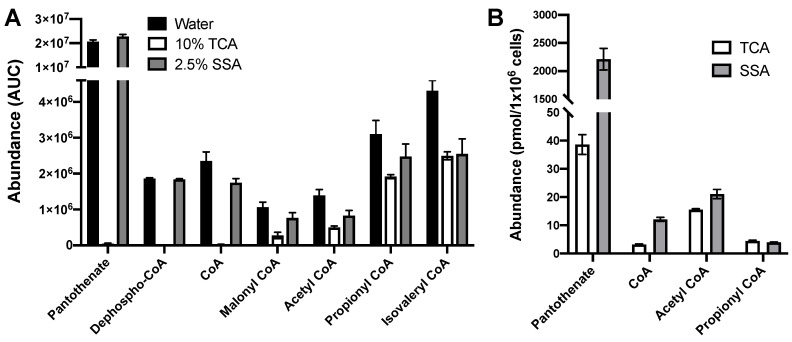
SSA-based sample preparation results in higher recovery of CoA biosynthetic pathway intermediates and short-chain acyl CoAs. (**A**) 1 nmol of each analyte was extracted with 200 μL of 10% (*w*/*v*) TCA followed by solid-phase extraction, 2.5% (*w*/*v*) SSA, or spiked into 200μL water. The following percent recoveries were obtained relative to water and shown as TCA vs. SSA: pantothenate (0% vs. >100%); dephospho-CoA (0% vs. >99%); CoA (1% vs. 74%); malonyl CoA (26% vs. 74%); acetyl CoA (36% vs. 59%); propionyl CoA (62% vs. 80%); and isovaleryl CoA (58% vs. 59%). (**B**) Metabolites were extracted from HEK 293FT cells using either direct extraction with SSA or TCA followed by solid-phase extraction. All data are presented as mean ± standard error of the mean (S.E.M.) for *n* ≥ 3 technical replicates.

To test whether TCA followed by SPE was a suitable extraction technique, 1 nmol of standards for pantothenate, dephospho-CoA, and each acyl CoA were extracted with 200 μL of 10% (*w*/*v*) TCA. TCA was removed by SPE and samples were reconstituted in 2.5% SSA prior to LC-MS/MS analysis [16,21,22]. For comparison, 1 nmol of each standard was also spiked into 200 μL of water and similarly analyzed. Relative recovery was determined by comparing the area under the curve (AUC) of each analyte extracted with TCA with the analyte in water. While using TCA demonstrated moderate recovery of acetyl CoA and CoA species with longer acyl tails, there was a marked decrease in recovery of free CoA and the biosynthetic intermediates (Figure 5A).

In the search for alternatives, we noted that SSA, in addition to being a commonly used solvent for reconstituting acyl CoAs for LC-MS/MS [16,21,22], has also been used for deproteinizing biological samples [30,31]. We determined that 2.5% (*w*/*v*) SSA fully deproteinized biological samples without lowering the pH of the extraction solution below 1.0, the recommended lower threshold for the HPLC column used in this study. Indeed, the extraction with 2.5% SSA exhibited a similar recovery of hydrophobic short-chain acyl CoAs relative to TCA. Additionally, we observed an increased recovery of free CoA and the CoA biosynthetic precursors of pantothenate and dephospho-CoA (Figure 5A). In terms of SSA, 2.5% was also able to efficiently extract these metabolites from the HEK 293FT human embryonic kidney cell line and compared favorably to TCA (Figure 5B). The results demonstrate that SSA is a preferred extraction solvent for our optimized LC-MS/MS method and obviates the need for solid phase extraction for these metabolites.

### 2.5. The Sample Preparation and LC-MS/MS Method Demonstrates the Minimal Matrix Effect and Preserves Accuracy

Having demonstrated that 2.5% SSA is a suitable extraction solvent, we then determined if our method displayed minimal variability due to matrix effects across the range of analytes. A matrix effect was determined as the percentage ratio of the AUC of analyte that had been spiked into the matrix versus the LC-MS/MS AUC of analyte that had been spiked into the extraction solution alone (Supplemental Table S2). For each analyte apart from dephospho-CoA, the matrix effect was marginal and exhibited less than a 10% ion suppression. Dephospho-CoA exhibited a consistently greater matrix effect, with an ion suppression of an average of 19% across its low, medium, and high spikes into the matrix.

Comparative accuracy was determined as the percent ratio of the mean baseline-subtracted concentrations for the spikes into the post-extraction matrix compared to mean concentrations of spikes into the extraction solution alone. In general, the internal standard-corrected matrix effect was deemed acceptable if it produced comparative accuracy within 85%. All analytes including dephospho-CoA achieved this average comparative accuracy. As such, despite the relatively large matrix effect of dephospho-CoA, this was partially compensated for by the internal standard when determining the concentration.

### 2.6. The Method Exhibits Acceptable Precision and Accuracy Parameters for Measured Analytes Across Their Entire Linear Ranges

The precision and accuracy for the sample preparation and LC-MS/MS method were determined according to guidelines provided by the FDA [32] and EMA [33] (Table 4). Low, medium, and high amounts of eight analytes were added to cell pellets, and the concentration was determined. Precision was reported as the relative standard deviation or coefficient of variation (CV). In general, precision was deemed acceptable with 15% ≥ CV and all metabolites considered fit this criterion. Accuracy was determined by comparing the average observed concentration to the amount spiked at that concentration after baseline correction. Accuracy was deemed acceptable when mean values were within 15% of the nominal values, and all metabolites considered met this mark.

### 2.7. The Method Detects Expected Perturbations in Levels of CoA and CoA Esters

**Figure 6:**
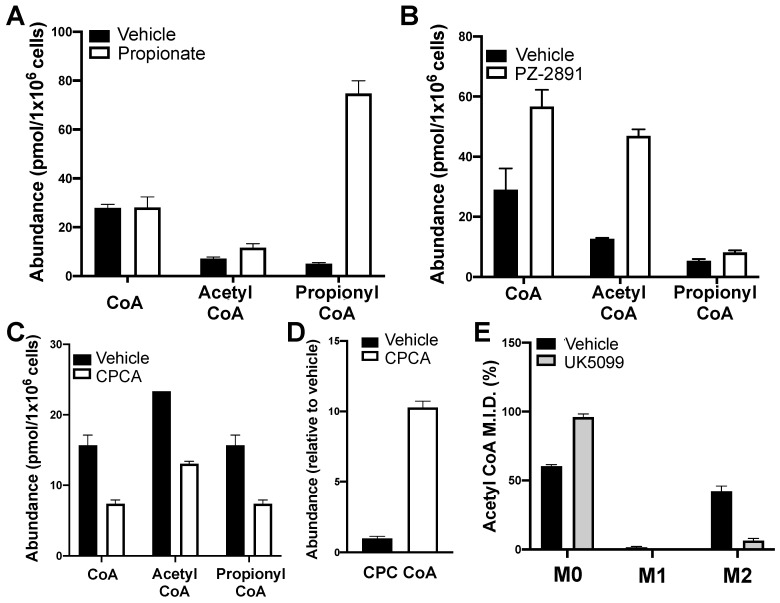
The sample preparation and method successfully detects expected perturbations in CoA metabolism: (**A**–**D**) HepG2 cells were treated for 24 h. with 1 mM propionate (**A**), 10 μM PZ-2891 (**B**), or 1 mM cyclopropanecarboxylic acid (CPCA; **C**,**D**). CoA and short-chain acyl CoA esters were extracted and measured as described above. (**E**) Mass isotopologue distribution (M.I.D.) of acetyl CoA extracted from HepG2 cells treated for 24 h. with U-^13^C_6_-glucose. All data are presented as mean ± standard error of the mean (S.E.M.) for *n* = 3 technical replicates.

We next validated our method by assessing acyl CoA levels in cells following treatments previously shown to increase and decrease steady-state levels of CoA-related metabolites. To determine if we could detect an enhanced synthesis of specific acyl CoAs, we treated HepG2 cells with 1 mM propionate. Consistent with prior reports [16], we observed a more than ten-fold increase in propionyl CoA levels following treatment (Figure 6A). We then measured whether the method could detect an increase in CoA synthesis with PZ-2891, a pantazine compound that is effective in treating preclinical models of pantothenate kinase-associated neurodegeneration (PKAN) [34]. The drug increases CoA levels by stabilizing pantothenate kinase in its active conformation. Indeed, HepG2 cells treated with 10 μM PZ-2981 caused an expected two-fold increase in free CoA in addition to an increased abundance of both acetyl and propionyl CoA (Figure 6B).

Having demonstrated that the method can detect increases in steady-state CoA levels, we then measured whether it can similarly detect decreased levels of CoA and CoA esters. We treated HepG2 cells with cyclopropanecarboxylic (CPCA), which is known to decrease CoA levels in mammalian hepatocytes [35]. Indeed, CPCA treatment halved intracellular free CoA levels and reduced the levels of short-chain acyl CoAs including acetyl and propionyl CoA (Figure 6C). As expected, cyclopropanecarboxyl-CoA levels were increased (Figure 6D), suggesting that formation of this acyl-CoA is likely associated with the depletion of free CoA. Finally, we determined if our method could be used to measure incorporation of isotopically labeled substrates into short-chain acyl CoAs. Treating HepG2 cells with uniformly labelled ^13^C_6_-glucose exhibited incorporation into acetyl CoA (“M2” isotopologue) that was substantially decreased in response to UK5099 [36], a potent and specific inhibitor of mitochondrial pyruvate uptake (Figure 6E).

### 2.8. The Method Detects Changes in the CoA Biosynthetic Pathway and Short-Chain Acyl CoA Species in IL-4-Polarized Macrophages Treated with Excess Etomoxir

**Figure 7:**
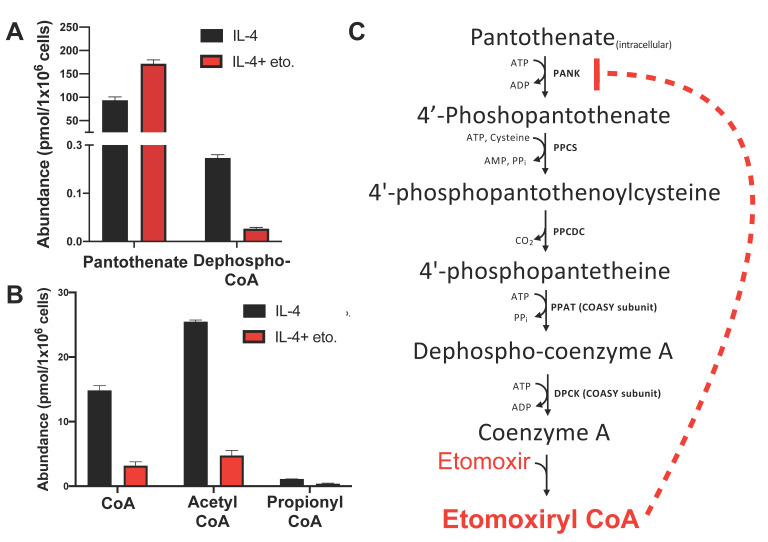
The SSA sample preparation and LC-MS/MS method detects changes in CoA synthesis pathway intermediates and short-chain acyl CoA species in IL-4-polarized macrophages following etomoxir treatment. Data are presented as mean ± standard error of the mean (S.E.M.) for *n* = 3 technical replicates.

Finally, we used our method to follow-up previous studies demonstrating the macrophage response to the anti-inflammatory cytokine IL-4 is inhibited by high concentrations of etomoxir. Treating macrophages with 200 μM etomoxir depleted free CoA (as measured by colorimetric assay) and qualitatively increased levels of pantothenate (measured separately by LC-MS/MS) [12]. We previously speculated high concentrations of etomoxir may be sufficient to deplete steady-state CoA levels by formation of an etomoxiryl CoA ester [12]. However, the ability to accurately quantify pantothenate, dephospho-CoA, and short-chain CoA esters in the same method allowed us to test the hypothesis that the etomoxiryl CoA ester may inhibit pantothenate kinase (PANK) similarly to feedback inhibition by other long-chain CoA esters [37]. Consistent with previous work, treatment with 200 μM etomoxir increased intracellular pantothenate levels (Figure 7A) and decreased steady-state free CoA levels (Figure 7B). Additionally, our LC-MS/MS method also revealed a reduction in dephospho-CoA (Figure 7A), supporting a mechanism whereby etomoxir depletes free CoA in part by PANK inhibition (Figure 7C). We also observed a reduction in other short-chain acyl CoAs in macrophages, notably acetyl CoA (Figure 7B), highlighting that the etomoxir-induced disruption of CoA homeostasis in macrophages perturbs multiple aspects of short-chain CoA biology.

## 3. Discussion

Here we detail an LC-MS/MS method that allows for the sensitive detection of short-chain acyl CoAs and metabolites in the CoA biosynthetic pathway. Our method efficiently overcomes limitations that otherwise preclude the use of a single method to analyze CoA esters and biosynthetic precursors. In particular, use of SSA circumvents the need for solid phase extraction (SPE) [17,31] as this single solvent can be used as both a deproteinizing agent and solvent for the reconstitution of acyl CoAs.

There are multiple advantages gained from bypassing SPE with the use of SSA. SPE results in the poor recovery of free CoA and biosynthetic intermediates. Although the divergent polarities between the two classes of analytes can lead to poor chromatography, this can be corrected by carefully adjusting the pH, including an ion pairing agent, and using an ultra-high performance liquid chromatography column. Eliminating SPE also circumvents the need to run multiple internal standards to correct for the differential recovery of various CoA esters. As many of these are not commercially available, intensive and specialized techniques—such as generation of SILEC-labeled internal standards—are often required [21,22]. The single, SSA extraction step, however, allows for the use of a single commercially available internal standard (crotonyl CoA). We demonstrate that endogenous levels of crotonyl CoA range from below 1% (cultured cells) to 3% (5 mg murine liver) of the internal standard, indicating that changes in endogenous crotonyl CoA levels would introduce minimal error when quantifying short-chain CoAs and CoA biosynthetic intermediates. However, caution should be exercised when interpreting data in model systems where steady-state crotonyl CoA levels are high or may substantially change between experimental groups, and SILEC-generated standards may be necessary.

Proof-of-concept biological experiments demonstrate that the proposed method is sensitive and can be used to quantify CoA-related metabolism in biological samples. In addition to establishing that this method can be used to detect both augmentation and depletion of acyl CoAs following pharmacological perturbations, the simultaneous quantification of metabolites involved in CoA biosynthesis may have biological and clinical utility. For example, more than 50% of neurodegeneration with brain iron accumulation (NBIA) cases are caused by dysfunction of enzymes involved in CoA synthesis [9,38]. Patient fibroblasts and tissues from preclinical disease models are often characterized by decreased free CoA [39,40], though mutations in both pantothenate kinase (PANK) or coenzyme A synthase (COASY) can cause NBIA [9,38]. As such, a combined LC-MS/MS method detecting short-chain acyl CoAs as well as the reactants and products of enzymes involved in NBIAs may be useful in better identifying the molecular basis resulting in these pathologies.

This method also proved useful in better understanding how etomoxir perturbs intracellular CoA homeostasis in IL-4-activated macrophages [12]. We were able to advance previous work by demonstrating that excess concentrations of etomoxir increased intracellular pantothenate levels while dephospho-CoA was decreased. This finding suggests that etomoxir may disrupt CoA metabolism by inhibiting a process downstream of pantothenate uptake but upstream of dephospho-CoA production. Given that long-chain acyl CoAs are potent inhibitors of PANK [37,41], it is likely that etomoxir is converted to its CoA thioester etomoxiryl CoA to inhibit PANK and block CoA biosynthesis. This reduction in steady-state free CoA in BMDMs is also associated with a reduction in short-chain acyl CoAs such as acetyl CoA, propionyl CoA, and succinyl CoA. As these metabolites are involved in many facets of cell physiology including the Krebs cycle, sterol synthesis, post-translational modifications, and epigenetic reprogramming, the mechanism linking disrupted CoA homeostasis by etomoxir with a reduced IL-4 response remains unclear.

## 4. Materials and Methods

All animal procedures were performed in accordance with the NIH Guide for the Care and Use of Laboratory Animals and were approved by the UCLA Animal Research Committee (ARC) under protocol #2020-027-1.

### 4.1. Materials and Reagents

DMEM (Cat#11965) was purchased from ThermoFisher (Waltham, MA, USA). Oasis HLB SPE columns were purchased from Waters (Milford, MA, USA). 5-sulfosalicylic acid, etomoxir, pantothenate, dephospho-CoA, and acyl CoA standards were purchased from Sigma-Aldrich (St. Louis, MO, USA). (E)-but-2-enoyl Coenzyme A (crotonoyl CoA) was purchased from Avanti Polar Lipids (Alabaster, Alabama, USA).

### 4.2. Cell Lines and Culture

HepG2 cells were obtained from American Type Culture Collection (ATCC) and cultured according to ATCC recommendations. Cells were maintained in MEM with 10% (*v*/*v*) FBS and 1% (*v*/*v*) penicillin/streptomycin. For biological validation experiments, 3.0 × 10^6^ cells were seeded in 10 cm dishes. After 48 h., cells were treated with 1 mM sodium propionate (Sigma-Aldrich), 1 mM cyclopropanecarboxylic acid (Sigma-Aldrich), 10 μM PZ-2891 (MedChem Express), or 5 μM UK5099 (Sigma-Aldrich) for 24 h. and harvested for extractions. The 293FT cell line was obtained from Invitrogen and maintained in DMEM with 10% FBS and 1% penicillin/streptomycin. For acyl CoA extractions, 2.0 × 10^6^ cells were seeded in 10 cm dishes and harvested after 72 h. Bone marrow-derived macrophages (BMDMs) were harvested by flushing the tibiae and femurs of 8–12-week male mice. Following red blood cell lysis, cells were centrifuged at 364× *g* for 5 min and resuspended in DMEM supplemented with 10% (*v*/*v*) FBS, 1% (*v*/*v*) penicillin/streptomycin, and a 5% (*v*/*v*) CMG-conditioned medium [42]. BMDMs were differentiated for 6 days and 5.0 × 10^6^ BMDMs were then re-plated in 10 cm dishes. After 48 h., new medium containing 20 ng/mL IL-4 (Peprotech) alone or IL-4 with 200 μM etomoxir was added to cells for 24 h. before extraction.

#### 4.2.1. Snap-Freezing of Cell Pellets

Cells in 10 cm plates were washed with 5 mL of phosphate buffered saline (PBS) and harvested by scraping in 1 mL of PBS before being transferred to 1.5 mL microfuge tubes. The cells were centrifuged at 1500× *g* at 4 °C for 5 min. The supernatant was removed and cell pellets were snap-frozen by with liquid nitrogen.

#### 4.2.2. Extraction of Pathway Intermediates and CoA Species from Cell Pellets with 5-Sulfosalicylic Acid (SSA) or Trichloroacetic Acid (TCA)

For SSA extraction, a 2.5% (*w*/*v*) SSA solution with 1 μM crotonoyl CoA was prepared. An extraction solution of 200 μL was added to cell pellets on wet ice and mixed. The samples were then centrifuged at 18,000× *g* for 15 min. Following centrifugation, supernatants were removed and transferred to glass LC-MS vials for analysis.

For extraction with 10% (*w*/*v*) TCA, a solution containing 1 μM crotonoyl CoA was prepared. An extraction solution of 200 μL was added to cell pellets and samples were resuspended by gentle pipetting. Solid phase extraction was then performed according to published methods [16,21,22]. Briefly, Oasis HLB columns (Waters) were first conditioned with 1 mL methanol, then equilibrated with 1 mL H_2_O. After equilibration, TCA-extracted samples were placed onto columns, washed with 1 mL H_2_O, and eluted with 1 mL of 25 mM ammonium acetate in methanol. Samples were dried overnight at 4 °C in a Centrivap benchtop vacuum concentrator (Labconco; St. Louis, MO, USA). Evaporated samples were reconstituted with 2.5% SSA and moved to LC-MS vials for analysis.

### 4.3. ESI LC-MS/MS Analytical Method

#### 4.3.1. Liquid Chromatography

Chromatography was performed on an Agilent 1290 ultra-high performance liquid chromatography (UHPLC) system using a Phenomenex Kinetex UHPLC C18 column (2.6 μm particle size, 2.1 mm ID × 150 mm). For analysis, 10 μL of sample was injected per run with a flow rate of 300 μL/min. The chromatography settings are detailed in Table 2.

#### 4.3.2. Mass Spectrometry

Mass spectrographic analysis was performed on a SCIEX 5500 QTrap run in both positive and negative modes and controlled by Analyst 1.6.2 software. Two separate MRM transitions were determined for each compound, and DP, EP, CE, and CXP for each compound were all manually optimized by tuning on direct infusions of approximately 100 ng compound per mL of 35/65 solvents A and B (see Figure 2A and Supplemental Table S1 for final settings). The source settings were optimized by tuning on a mix of analytes while also flowing in 35/65 solvents A and B at 300 uL/min with temperature set at 450 °C; GS1 and GS2 at 45; curtain gas at 35; CAD gas at medium; ion spray voltage in positive mode = 5500 volts; and ion spray voltage in negative mode = –4500 volts.

#### 4.3.3. Data Analysis

Peaks were integrated and concentrations were calculated using MultiQuant software (SCIEX). The concentration of each analyte was determined from the ratio of the analyte to its internal standard by a calibration curve. Curves were generated in an extraction solution and a linear regression with 1/x weighting was performed for each curve to determine the line of best fit. Data for biological samples was normalized to cell number.

### 4.4. Validation of Sample Preparation and LC-MS/MS Methods

Validation was performed in accordance with both the FDA guidelines for bioanalytical method validation [32] and the European Medicines Agency guidelines on bioanalytical method validation [33,43].

#### 4.4.1. Linearity, Lower Limit of Detection (LLOD), and Lower Limit of Quantitation (LLOQ)

Calibration standards were spiked into the extraction solution across a wide range of concentrations involving at least 6–8 concentrations, and an internal standard was added to all calibrant solutions at the concentration matching biological samples. The allowable bias was within 15% for all calibrations for ≥75% of the standards except at the LLOQ, for which the allowable bias was within 20%. Linearity was determined by the correlation coefficient of the linear regression with 1/x weighting. LLOQ was determined as the lowest value in the calibration range for which bias was within 20%. LLOD was determined as the lowest value in the concentration range for which the peak height of the analyte chromatogram was five-fold greater than the baseline signal.

#### 4.4.2. Matrix Effect and Comparative Accuracy

Approximately 2.0 × 10^7^ 293FT cells were divided into 20 equal pellets that were extracted in the SSA as above. Standards were then spiked into groups of 5 post-extraction matrix samples at 0 (0 pmol), low (62.5 pmol), medium (250 pmol) and high (1000 pmol) levels. Low, medium, and high standards were also spiked into equal volumes of the extraction solvent (*n* = 5 per group) and thus there were 7 separate groups of 5 replicates each. The group average of the AUC for each analyte in each group was determined. To determine the matrix effect, the baseline mean AUC for each analyte in post-extraction matrix (0 pmol spike group) was first subtracted from the mean AUC for each analyte in the low, medium, and high spikes into post-extraction matrix. These baseline-subtracted means were then compared to the mean AUC for each analyte in the low, medium, and high spikes in the extraction solvent. The matrix effect was reported as the percentage-based ratio of this comparison. Following the determination of the matrix effect upon AUC, analyte concentrations were determined for all groups. Comparative accuracy was determined as the percentage-based ratio of the mean baseline-subtracted concentrations for the spikes in the post-extraction matrix relative to the mean concentrations for those spikes in the extraction solution. In general, the internal standard corrected matrix effect was deemed acceptable if it produced comparative accuracy of within 15%.

#### 4.4.3. Precision and Accuracy

Approximately 2.0 × 10^7^ 293FT cells were divided into 20 equal pellets that were extracted in the SSA as above. Low (62.5 pmol), medium (250 pmol), or high (1000 pmol) amounts of all eight core standards in addition to controls lacking standards were spiked into groups of 5 pellets. The SSA extraction solution was added to all 20 samples and the concentration was calculated for each analyte for each sample. Precision was determined for each analyte across the 5 replicates at each concentration (0, 62.5, 250, and 1000 pmol) as the relative standard deviation or coefficient of variation (CV). In general, precision was deemed acceptable with CV ≤ 15%. Accuracy was determined by comparing the average calculated concentration for each analyte at each spiked concentration to the nominal value, which was the amount spiked at that concentration added to the value determined for the baseline or 0 group. In general, accuracy was deemed acceptable when mean values were within 15% of the nominal values.

## Figures and Tables

**Table 1:** Parent and daughter ion m/z for short-chain acyl CoAs, dephospho-CoA, and pantothenate.

**Compound** **Name**	**Acyl Group Formula**	**Acyl Group** **Structure**	**Parent (m/z)**	**Daughter 1** **(m/z)**	**Daughter 2** **(m/z)**
CoA	H	H	768.1	261.1	428.1
Acetyl CoA	COCH_3_	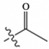	810.1	303.1	428.1
Propionyl CoA	COCH_2_CH_3_	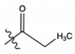	824.1	317.1	428.1
Isovaleryl CoA	COCH_2_(CH_3_)_2_	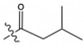	852.1	345.1	428.1
Malonyl CoA	COCH_2_CO_2_H	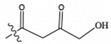	854.1	347.1	428.1
Succinyl CoA	CO(CH_2_)_2_CO_2_H	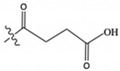	868.1	361.1	428.1
**Compoud** **Name**	**Chemical Formula**	**Compound** **Structure**	**Parent (m/z)**	**Daughter 1** **(m/z)**	**Daughter 2 (m/z)**
Pantothenate	C_9_H_17_NO_5_	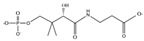	218.0	88.0	145.8
Dephospho-CoA	C_21_H_35_N_7_O_13_P_2_S	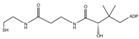	688.1	261.1	348.1

**Table 2:** The following solvents were used during the chromatography: Solvent A, 5 mM ammonium acetate + 2.5 mM DMBA (pH 5.6) and Solvent B, 95% acetonitrile, 5% H_2_O + 5 mM ammonium acetate.

Time (min)	%A	%B	Notes
0	98	2	Divert to waste (0–5.5 min)
1.5	98	2	
9	50	50	Divert to MS (5.5–10.5 min)
9.5	2	98	
14.5	2	98	Divert to waste (10.5–22 min)
15	98	2	
22	98	2	

**Table 3:** The lower limits of detection (LLOD) and quantitation (LLOQ) are calculated as described in the text.

Compound	Linear Regression	r	LLOD (pmol)	LLOQ (pmol)
Pantothenate	Y = 0.00264x + 0.0307	0.98	1	7.4
Dephospho-CoA	Y = 0.00481x + 0.0892	0.97	0.4	3.7
CoA	Y = 0.00546x − 0.0196	0.97	1	3.7
Malonyl CoA	Y = 0.00221x − 0.00851	0.99	3	3.7
Succinyl CoA	Y = 0.00095x − 0.0011	0.95	1	7.4
Acetyl CoA	Y = 0.00461x − 0.0163	0.97	1	3.7
Propionyl CoA	Y = 0.00660x + 0.0367	0.99	2	3.7
Isovaleryl CoA	Y = 0.00864x − 0.1853	0.99	1	7.4

**Table 4:** The precision and accuracy are determined as described in the text. NA is defined as not applicable.

	Standards (pmol)	Calculated Concentration (pmol/1 × 10^6^ cells)	SEM	Precision CV (%)	Accuracy (% of Nominal Value)
Pantothenate	0	969.15	7.74	1.60	NA
	62.5	1231.50	48.44	7.87	119.37
	250	1421.00	78.83	11.10	116.56
	1000	2244.50	31.47	2.80	113.98
Dephospho-CoA	0	1.68	0.08	9.78	NA
	62.5	57.39	4.78	16.64	89.41
	250	228.81	23.02	20.12	90.91
	1000	961.96	24.63	5.12	96.03
CoA	0	49.98	4.67	18.68	NA
	62.5	143.03	4.81	6.73	127.16
	250	277.50	16.61	11.97	92.51
	1000	865.48	7.02	1.62	82.43
Acetyl CoA	0	65.67	1.81	5.52	NA
	62.5	127.05	8.85	13.94	99.13
	250	356.48	14.96	8.40	112.93
	1000	1402.79	32.15	4.58	131.63
Propionyl CoA	0	7.95	0.20	5.03	NA
	62.5	75.03	5.98	15.93	106.49
	250	277.52	4.06	2.92	107.59
	1000	1132.35	59.58	10.52	112.34
Isovaleryl CoA	0	3.17	0.21	13.19	NA
	62.5	67.53	6.33	18.76	102.82
	250	314.52	4.34	2.76	124.23
	1000	1383.19	50.47	7.30	137.88
Malonyl CoA	0	2.45	0.18	14.84	NA
	62.5	62.03	2.94	9.48	95.50
	250	265.98	20.15	15.15	105.36
	1000	1077.85	69.21	12.84	107.52
Succinyl CoA	0	208.61	11.79	11.31	NA
	62.5	295.38	22.62	15.32	108.96
	250	507.42	25.24	9.95	110.64
	1000	1279.86	71.49	11.17	105.90

## Data Availability

Data is available from the corresponding authors upon request.

## Supplementary Materials

The following are available online at https://www.mdpi.com/article/10.3390/metabo11080468/s1, Figure S1. The CoA biosynthetic pathway with corresponding structures, Figure S2. Pantothenate is only detectable with adequate sensitivity with negativemode MRM, Figure S3. Free CoA is produced in the ESI source from acetyl, propionyl, and isovaleryl CoA, Figure S4. Endogenous crotonoyl CoA concentrations, Table S1. Mass spectrometry parameters for detection of short-chain acyl CoAs, dephospho-CoA, and pantothenate, Table S2. Determination of matrix effect.
