# Supplementary material for: A Single LC-MS/MS Analysis to Quantify CoA Biosynthetic Intermediates and Short-Chain Acyl CoAs
Source: Metabolites. 2021 Jul 21;11(8):468. doi: 10.3390/metabo11080468 (PMC8401288; doi:10.3390/metabo11080468)
Supplement: Supplementary file 1 [file metabolites-11-00468-s001.zip › metabolites-1244765-supplementary.pdf]

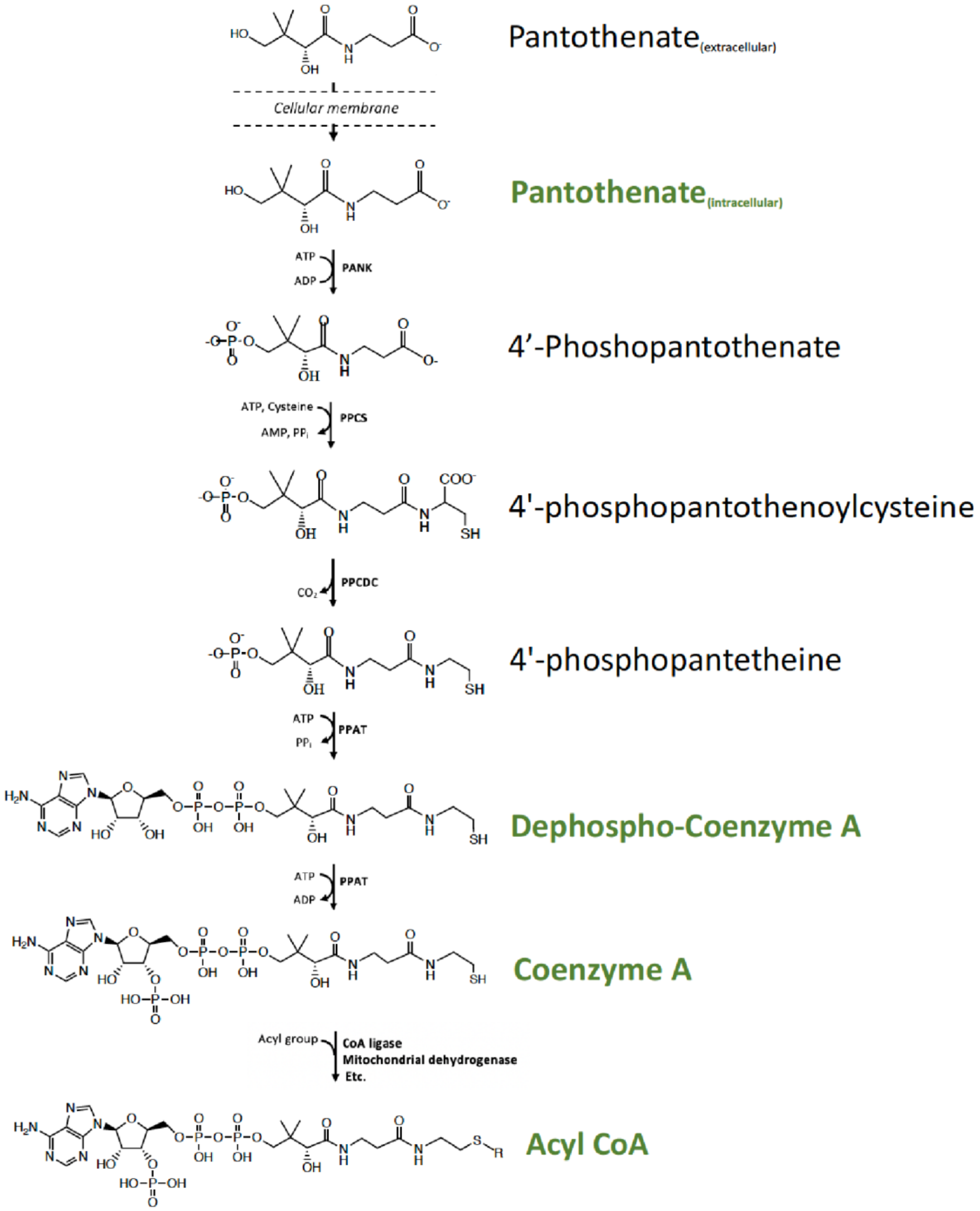

Figure S1. The CoA biosynthetic pathway with corresponding structures: As in Figure 1, the metabolites detected by the method detailed in this manuscript are highlighted in green. PANK, pantothenate kinase; PPCS, phosphopantothenoylcysteine synthase; PPCDC, phosphopantothenoylcysteine decarboxylase; PPAT, phosphopantetheine adenylyl transferase; DPCK, dephosphocoenzyme A kinase. The bifunctional enzyme Coenzyme A synthase (COASY) is comprised of PPAT and DPCK.

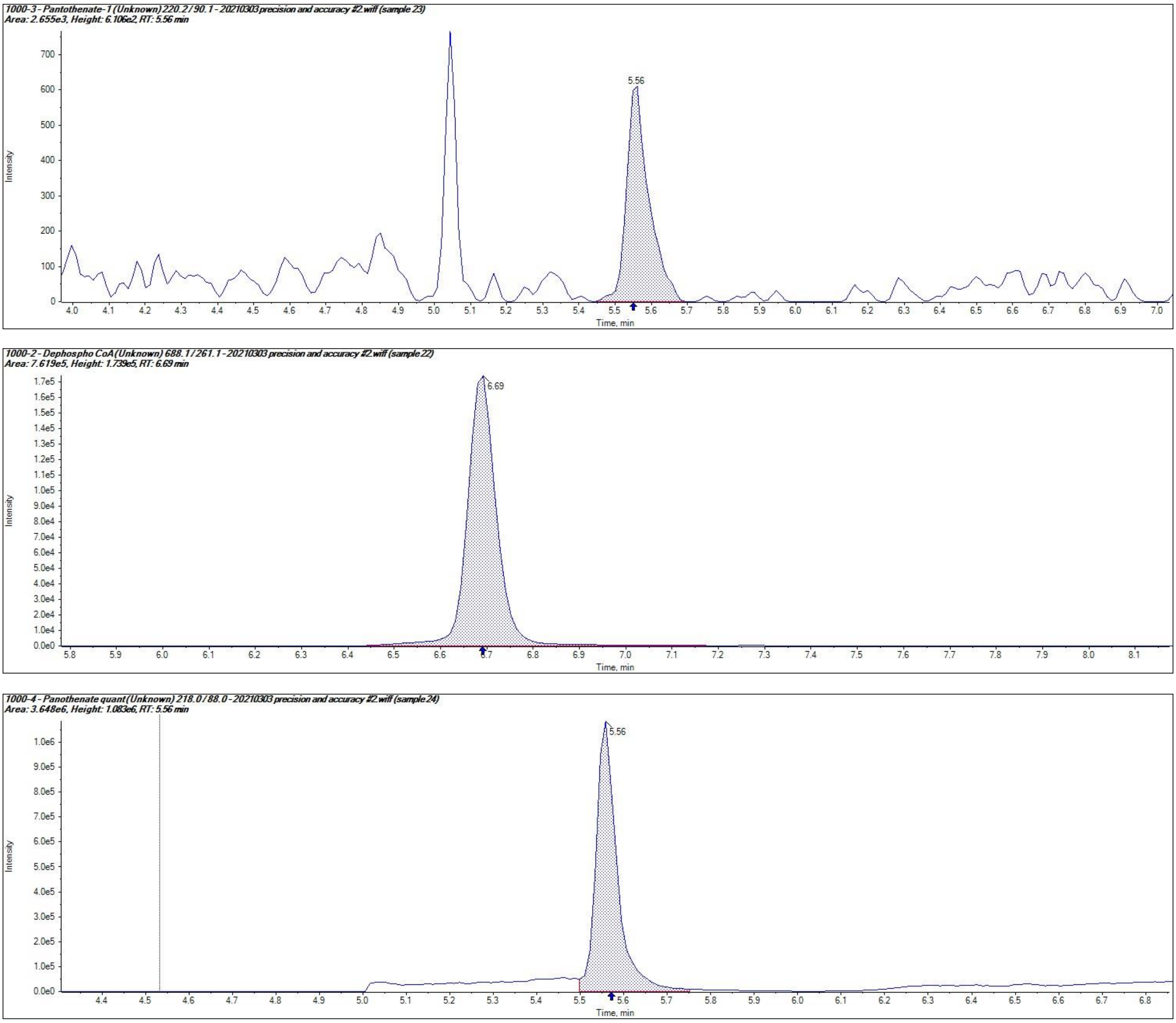

Figure S2. Pantothenate is only detectable with adequate sensitivity with negativemode MRM: (*Top*) Pantothenate analyzed with positive-mode MRM. Even with optimized MRM settings, the signal is barely above the lower limit of detection (5X the noise floor). (*Middle*) Dephospho-CoA detected with positive-mode MRM with 200-fold more intensity than pantothenate. (*Bottom*) Pantothenate detected with negative-mode MRM with $1x10^3$ the signal intensity of positive-mode. All analytes were measured at 5 pmol/μL.

Table S1. Mass spectrometry parameters for detection of short-chain acyl CoAs, dephospho-CoA, and pantothenate.

Notes	Q1 Mass (Da)	Q3 Mass (Da)	Dwell time (msec)	ID	DP (volts)	EP (volts)	CE (volts)	CXP (volts)
Core positive mode MRM settings								
	810.1	303.1	25	Acetyl CoA-quantitative	100	6	40	11
	810.1	427.9	25	Acetyl CoA-qualitative	100	6	36	14
	768.1	261.1	25	CoA-SH-quantitative	80	8	36	15
	768.1	428.2	25	CoA-SH-qualitative	80	8	36	15
	824.1	317.1	25	Propionyl CoA-quantitative	100	6	40	11
	824.1	428	25	Propionyl CoA-qualitative	100	6	36	14
	868.1	361.3	25	Succinyl CoA-quantitative	160	8	45	15
	868.1	428.2	25	Succinyl CoA-qualitative	160	8	40	16
	854.1	347.1	25	Malonyl CoA-quantitative	130	8	38	15
	854.1	428	25	Malonyl CoA-qualitative	130	8	39	15
	688.1	261.1	25	Dephospho CoA	100	6	40	15
	836.1	329.1	25	Crotonoyl CoA-quantitative	100	6	40	10
	836.1	428	25	Crotonoyl CoA-qualitative	100	6	36	10
	852.1	345.1	25	Isovaleryl CoA -quantitative	40	8	41	10
	852.1	428.2	25	Isovaleryl CoA-qualitative	40	8	36.5	16
Core negative mode MRM settings								
	218	88	25	Pantothenate-quantitative	-100	-10	-20	-15
	218	145.8	25	Pantothenate-qualitative	-100	-10	-20	-15
Additional positive mode MRM settings								
*	836.2	329.1	25	Cyclopropanecarboxyl-CoA	100	6	40	13
#	811.1	304.1	20	Acetyl CoA -M+1	100	6	40	11
#	812.1	305.1	20	Acetyl CoA -M+2	100	6	40	11
#	813.1	306.1	20	Acetyl CoA-M+3	100	6	40	11

*** Additional positive mode settings for experiment represented in Figure 6B. # Additonal positive mode settings for experiment represented in Figure 6D.**

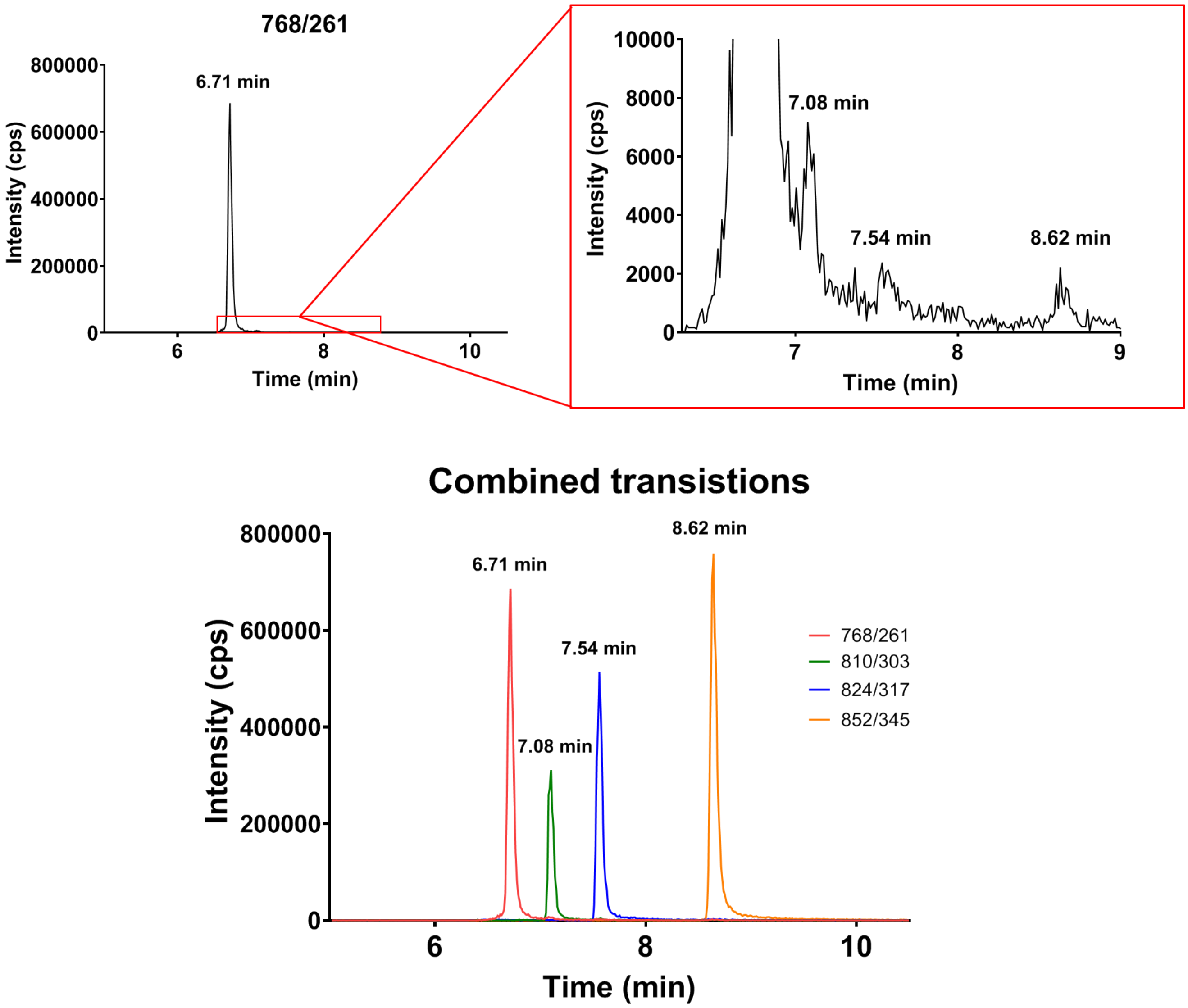

Figure S3. Free CoA is produced in the ESI source from acetyl, propionyl, and isovaleryl CoA: Standards were injected and the MRM channel associated with CoA (768/261) was monitored. Peaks of 768/261 were observed at 6.71 min, the retention time of CoA-SH *(top panel, left);* as well as at 7.08, 7.54, and 8.62 (*top panel, inset*). The retention times of 7.08, 7.54, and 8.62 were otherwise associated with dominant peaks for acetyl CoA (810/303), propionyl CoA (824/317), and isovaleryl CoA (852/345) (*bottom panel*).

Table S2. Determination of matrix effect

	Stds spiked in	Stds in post-extraction matrix (AUC)	Stds in extraction solvent (AUC)	Matrix effect (as % of stds in solvent)	Stds in postextraction matrix (pmol)	Stds in extraction solvent (pmol)	Comparative accuracy (as % of stds in solvent)
Pantothenate	0	2000200.00	NA		105.32	NA	
	62.50	2978250.00	1031450.00	94.82	182.39	57.04	135.11
	250.00	5579000.00	3958250.00	90.41	283.53	218.39	81.60
	1000.00	22342500.00	20840000.00	97.61	1156.25	1043.00	100.76
Dephospho CoA	0	0	NA		0.00	NA	
	62.50	30205.00	38385.00	78.69	63.40	77.09	82.25
	250.00	135900.00	156750.00	86.70	267.73	301.91	88.68
	1000.00	550125.00	682450.00	80.61	1098.18	1207.27	90.96
CoA -SH	0	75627.50	NA		81.13	NA	
	62.50	119425.00	51492.50	85.06	130.94	55.22	90.20
	250.00	286650.00	226825.00	93.03	288.80	242.39	85.68
	1000.00	1033325.00	1026500.00	93.30	1053.57	1009.71	96.31
Acetyl CoA	0	3271.00	NA		13.80	NA	
	62.50	14167.50	11860.50	91.87	73.65	59.98	99.77
	250.00	43103.33	40760.00	97.72	216.27	203.50	99.49
	1000.00	194200.00	219600.00	86.94	968.80	1078.55	88.54
Propionyl CoA	0	22780.00	NA		22.73	NA	
	62.50	86710.00	55222.50	115.77	80.58	50.80	113.85
	250.00	264150.00	226025.00	106.79	234.62	215.40	98.37
	1000.00	1036000.00	1143500.00	88.61	938.00	992.67	92.20
Isovaleryl CoA	0	3540.00	NA		3.87	NA	
	62.50	62860.00	58707.50	101.04	65.78	60.87	101.70
	250.00	278800.00	269425.00	103.48	269.16	277.80	96.89
	1000	1198000.00	1189400.00	100.72	1175.00	1134.14	103.60
Malonyl CoA	0	0	NA		0.00	NA	
	62.50	11617.50	10444.75	111.23	59.48	52.99	112.24
	250.00	48592.50	47407.50	102.50	226.30	239.18	94.62
	1000.00	225525.00	258450.00	87.26	1060.25	1177.55	90.04
Succinyl CoA	0	8135.75	NA		90.02	NA	
	62.50	12180.00	4675.00	86.51	162.20	64.24	112.35
	250.00	25990.00	19500.00	91.56	326.15	258.43	91.37
	1000.00	88582.50	74340.00	108.21	1089.95	883.25	113.21

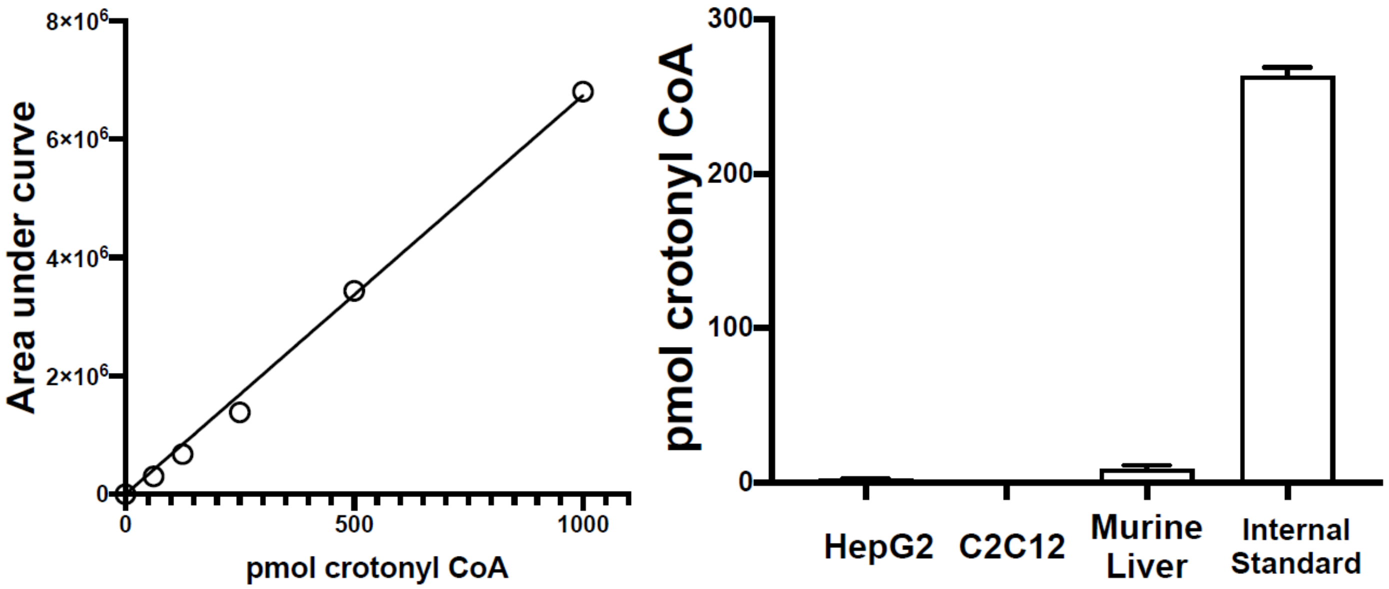

Figure S4. Endogenous crotonoyl CoA concentrations are significantly below the amount of crotonoyl CoA utilized as an internal standard for the proposed method. (*Left*). Increasing concentrations of crotonoyl CoA were spiked into 200μL of extraction solution and analyzed via LC-MS/MS. The calibration curve was generated by plotting the area under the curve for crotonoyl CoA against the amount of analyte contained in 200μL of extraction solution. (*Right*) The abundance of endogenous crotonoyl CoA was determined for $6x10^6$ HepG2 cells, $8x10^8$ C2C12 cells, and 5mg murine liver tissue and compared to a blank sample spiked with 1μM crotonoyl CoA as an internal standard. Cell lines had endogenously crotonyl CoA levels less than 1% of the internal standard, and less than 3% for murine liver. Data are presented as mean ± standard error of the mean (S.E.M.) for n^{3}2 technical replicates.
